# Atmosphere, ecology and evolution: what drove the Miocene expansion of C_4_ grasslands?

**DOI:** 10.1111/j.1365-2745.2007.01323.x

**Published:** 2008-01

**Authors:** Colin P Osborne

**Affiliations:** Department of Animal and Plant Sciences, University of Sheffield Sheffield S10 2TN, UK

**Keywords:** atmospheric CO_2_, C_4_ plants, climate change, fire, grassland, grazing, Poaceae, rainfall, savanna, seasonality

## Abstract

1. Grasses using the C_4_ photosynthetic pathway dominate today's savanna ecosystems and account for ∼20% of terrestrial carbon fixation. However, this dominant status was reached only recently, during a period of C_4_ grassland expansion in the Late Miocene and Early Pliocene (4–8 Myr ago). Declining atmospheric CO_2_ has long been considered the key driver of this event, but new geological evidence casts doubt on the idea, forcing a reconsideration of the environmental cues for C_4_ plant success.
2. Here, I evaluate the current hypotheses and debate in this field, beginning with a discussion of the role of CO_2_ in the evolutionary origins, rather than expansion, of C_4_ grasses. Atmospheric CO_2_ starvation is a plausible selection agent for the C_4_ pathway, but a time gap of around 10 Myr remains between major decreases in CO_2_ during the Oligocene, and the earliest current evidence of C_4_ plants.
3. An emerging ecological perspective explains the Miocene expansion of C_4_ grasslands via changes in climatic seasonality and the occurrence of fire. However, the climatic drivers of this event are debated and may vary among geographical regions.
4. Uncertainty in these areas could be reduced significantly by new directions in ecological research, especially the discovery that grass species richness along rainfall gradients shows contrasting patterns in different C_4_ clades. By re-evaluating a published data set, I show that increasing seasonality of rainfall is linked to changes in the relative abundance of the major C_4_ grass clades Paniceae and Andropogoneae. I propose that the explicit inclusion of these ecological patterns would significantly strengthen climate change hypotheses of Miocene C_4_ grassland expansion. Critically, they allow a new series of testable predictions to be made about the fossil record.
5. *Synthesis*. This paper offers a novel framework for integrating modern ecological patterns into theories about the geological history of C_4_ plants.

## Carbon dioxide and the expansion of C_4_ grasslands

**Fig. 1:**
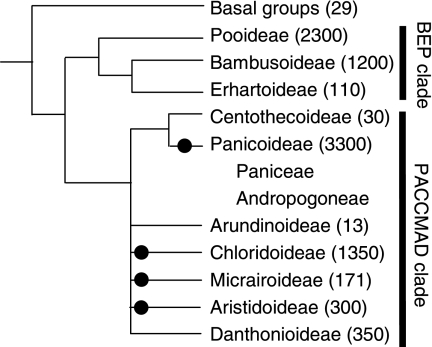
Cladogram displaying the hypothesized relationships among subfamilies of the Poaceae based on multiple markers, with the number of species shown for each, and filled circles (•) indicating the unequivocal origins of C_4_ photosynthesis in independent clades (Watson & Dallwitz 1992; GPWG 2001; Linder & Rudall 2005; Sánchez-Ken *et al*. 2007). Up to eight independent origination events may have occurred within the Panicoideae.

Major contrasts in the climatic preferences of grass subfamilies have been noted for more than half a century (Hartley 1950), but their significance was only recognized following the discovery of C_4_ photosynthesis. Tropical and subtropical grasslands are dominated by the predominantly C_4_ Panicoideae and Chloridoideae (Hartley 1958a, b; Hartley & Slater 1960), which together account for more than half of the world's grass species (Fig. 1; Linder & Rudall 2005) and ∼20% of gross terrestrial carbon fixation (Lloyd & Farquhar 1994). Their carbon-concentrating mechanism suppresses the energetically wasteful process of photorespiration that plagues C_3_ grasses at high temperatures, and significantly raises the efficiency of photosynthesis in warm climate regions. In contrast, C_3_ grass subfamilies such as the Pooideae are largely confined to temperate climates, where photorespiration is naturally limited by lower temperatures (Hartley 1961, 1973).

**Fig. 2:**
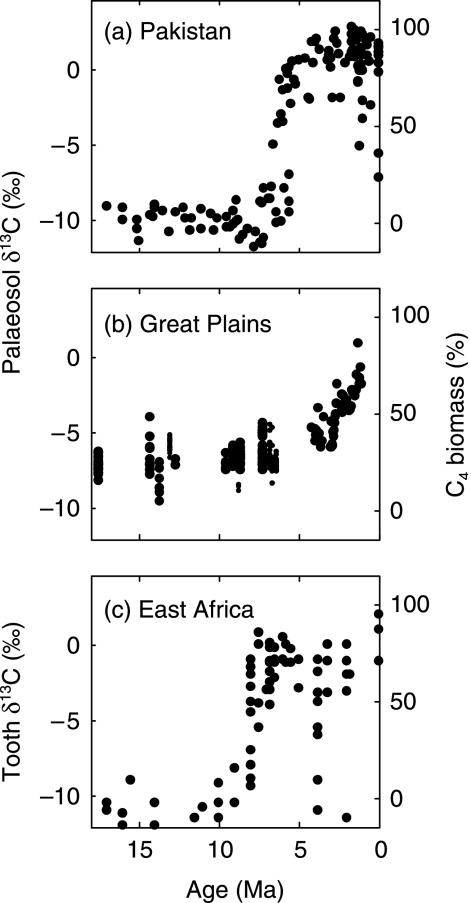
Examples of the shifts in stable carbon isotope ratio (δ^13^C) characterizing the Miocene rise of C_4_ plants in (a) Pakistan (Quade & Cerling 1995), (b) the Great Plains (Fox & Koch 2003) and (c) East Africa (Cerling *et al*. 1997). Values for (a) and (b) were obtained from palaeosol carbonates, and the proportion of biomass contributed by C_4_ plants calculated following Fox & Koch (2003). Values for (c) are from the tooth enamel of mammalian herbivores, with the proportion of C_4_ plants in their diets after Cerling *et al*. (1997).

The evolutionary history of these patterns was elucidated only in the past two decades, following the realization that C_4_ photosynthesis imparts a distinctive carbon isotope signature to plant materials and trophic pathways. By analysing the isotopic composition of fossilized soils (palaeosols) and the teeth of herbivores, geochemists uncovered a surprise; the domination of low-latitude ecosystems by C_4_ grasses is a recent phenomenon in geological terms, occurring only 4–8 Myr ago (Ma) at the Miocene–Pliocene boundary, when C_4_ grasslands expanded across at least four continents (Fig. 2; Quade *et al*. 1989; Cerling *et al*. 1997). The factors behind this evolutionary phenomenon have remained controversial since its discovery.

At first, debate focused on the relative merits of CO_2_ as a driver of C_4_ grassland expansion (e.g. Cerling *et al*. 1994; Morgan *et al*. 1994a, b). The case for CO_2_ was persuasive, and based on the premise that selective and competitive advantages of C_4_ photosynthesis result from the energetic benefits of eliminating photorespiration at high temperatures and low atmospheric CO_2_ (Ehleringer *et al*. 1991, 1997; Cerling *et al*. 1997). These advantages over the ancestral C_3_ condition are reversed when photorespiration is naturally suppressed by low temperatures or high CO_2_, because the C_4_ carbon-concentrating mechanism requires energy. This leads to critical thresholds of temperature (around 20–25 °C at today's atmospheric CO_2_ concentration) and CO_2_ (around 500 p.p.m. in tropical environments) where C_3_ and C_4_ photosynthesis have equal energy requirements and, by extension, equal competitive and selective advantages (Cerling *et al*. 1997). The proponents of this elegant hypothesis noted the close correspondence between the theoretical temperature threshold and mean growing season value in modern regions of equal C_3_ and C_4_ grass species richness, and postulated that declining CO_2_ crossed an equivalent threshold at the Miocene–Pliocene boundary (Ehleringer *et al*. 1991, 1997; Cerling *et al*. 1997). Their ideas were supported by a geochemical model of atmospheric CO_2_, which indicated the necessary decline during the Cenozoic (Berner 1998).

**Fig. 3:**
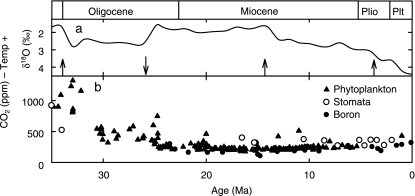
CO_2_ and climate change from the Oligocene to the present day. (a) Oxygen isotope values of deep-sea foraminifera (δ^18^O) displayed as a locally weighted running mean calculated by Zachos *et al*. (2001). This serves as a combined proxy for the global deep-sea temperature and continental ice volume, with increases in δ^18^O indicating cooling and ice growth. Periods of rapid ice sheet growth are indicated by ‘up’ arrows, and contraction by ‘down’ arrows. The geological timescale shows ages in Myr ago (Ma); Plio, Pliocene; Plt, Pleistocene. (b) CO_2_ proxy data based on carbon isotope ratios of marine phytoplankton, stomatal densities of fossil leaves and the boron isotope ratios of planktonic foraminifera compiled by Royer (2006). Palaeosol proxy data are not shown, because the uncertainty in this technique (± 500 p.p.m.) precludes meaningful estimation of low CO_2_ levels.

By the late 1990s, the ideas underpinning the CO_2_ starvation hypothesis were widely accepted, but still awaited the crucial test provided by palaeo-CO_2_ reconstructions. This soon came with the publication of three data sets (Pagani *et al*. 1999; Pearson & Palmer 2000; Royer *et al*. 2001), each using an independent proxy for CO_2_, and each showing a long period of stasis in the level of atmospheric CO_2_ during the expansion of C_4_ grasslands (Fig. 3b; reviewed by Royer 2006). Unless these palaeo-CO_2_ proxy records are challenged on technical or theoretical grounds, the geological evidence therefore stands firmly against the CO_2_ starvation hypothesis for C_4_ grassland expansion, and new mechanisms must be sought. Instead, the latest evidence suggests that atmospheric CO_2_ dropped sharply through the C_3_–C_4_‘crossover threshold’ at 25–30 Ma during the Oligocene, when it initiated our modern ‘icehouse’ era of advancing and retreating polar ice sheets (Fig. 3; Pagani *et al*. 2005; Royer 2006). The emerging picture of palaeoenvironmental change therefore lends credence to an alternative hypothesis, proposing declining CO_2_ concentration as a key selection pressure for the evolutionary origins of C_4_ photosynthesis in the grasses, rather than C_4_ grassland expansion (Ehleringer *et al*. 1991; Pagani *et al*. 2005).

Here, I review two major issues currently facing geologists, physiological ecologists and ecosystem scientists with interests in this field. I first evaluate the proposed role of CO_2_ in the evolutionary origins of C_4_ photosynthesis, focusing on the question of when the pathway first arose in the grasses. However, my principal focus is on the current debate surrounding the causes of C_4_ grassland expansion, particularly the hypothesized effects of climate change and fire. I argue that a deeper understanding of these proposed abiotic drivers could be achieved by explicitly considering their contrasting interactions within independent groups of C_4_ grasses.

## Dating the origins of C_4_ photosynthesis

A crucial test of the CO_2_ hypothesis for C_4_ grass origins is to establish whether the first appearance of the pathway coincides with the Oligocene drop in atmospheric CO_2_. Using a molecular clock, Gaut & Doebley (1997) calculated that divergence of two major C_4_ grass tribes, the Paniceae and Andropogoneae (Fig. 1), occurred 25–32 Ma, leading later authors to argue that origins of C_4_ photosynthesis must therefore be at least this old (e.g. Kellogg 1999). However, subsequent phylogenetic analysis cast doubt on this idea, suggesting that C_4_ origins may have post-dated the divergence event, evolving in up to eight independent subgroups of the Paniceae and Andropogoneae (Giussani *et al*. 2001; Aliscioni *et al*. 2003; Sage 2004). Dating the nodes of the grass phylogenetic tree (Fig. 1) has proved difficult because it is becoming increasingly clear that variation in the mutation rate among branches generates large errors in molecular clocks (Pulquiéro & Nichols 2007). Attempts at calibration using the fossil record have yielded dates that vary hugely because of the low sample sizes used (Linder & Rudall 2005). More precise dating of evolutionary events within the grass family therefore depends crucially on a better resolved fossil record.

Fossils only provide minimum dates for nodes of the phylogenetic tree because the geological record is incomplete. This problem is particularly acute for grasses of open habitats, where the potential for fossilization is extremely low (Cerling 1999). The oldest C_4_ grass macrofossils date to only 12.5 Ma in the Middle Miocene (reviewed by Osborne & Beerling 2006). However, the pollen record for grasses overall stretches back more than 65 Myr to the Late Cretaceous (Jacobs *et al*. 1999), indicating the potential for much earlier C_4_ origins. Grass pollen cannot be identified below the family level, but phytolith traits map onto the grass phylogeny at the subfamilial scale (Piperno & Sues 2005; Prasad *et al*. 2005). Phytoliths form, to a varying degree, in all groups of living vascular plants (Piperno 2006). However, grasses have a higher silica production than most other plants (Hodson *et al*. 2005), and phytoliths in this family are more diagnostic than for most other plant groups. The interpretation of fossil phytolith assemblages is complicated by multiplicity (each species produces more than one phytolith morphotype), and redundancy (different species produce similar morphotypes) (Piperno 1988). Nevertheless, the comparison of fossil assemblages with modern reference collections has allowed robust statistical inferences about the phylogenetic affinities and ecology of extinct grass communities (Strömberg 2004, 2005).

Phytolith analysis has been used to identify grasses with affinities to the PACCMAD crown group (Fig. 1) from 65- to 67-Myr-old dinosaur dung in India (Prasad *et al*. 2005), and from the predominantly C_4_ subfamily Chloridoideae (Fig. 1) in 19-Myr-old Great Plains sediments (Strömberg 2005). Although this technique cannot establish when C_4_ photosynthesis originated in each clade, complementary methods have been developed for analysing the carbon isotopic signature of phytolith assemblages (Smith & White 2004). Interpretation of these data is difficult because of interspecific variation and differences in the biochemical nature of carbon compounds between C_3_ and C_4_ grass phytoliths (reviewed by Smith & White 2004). However, preliminary data suggest that up to 50% of Great Plains grasses may have used the C_4_ pathway by 12 Ma (Smith 2001).

Geochemical analyses of herbivore teeth, palaeosols and the molecular markers of plant cuticles trace the C_4_ carbon isotope signal back to 16–18 Ma in the Early Miocene (reviewed in Tipple & Pagani 2007). These data are consistent with the presence of C_4_ grasses comprising up to 30% of the total biomass in tropical and subtropical ecosystems throughout the Miocene (Fox & Koch 2003; Pagani *et al*. 2005; Tipple & Pagani 2007). However, the use of bulk isotope analyses to estimate C_4_ biomass in extinct plant communities is not a precise science. Critical uncertainties remain about the isotopic composition of atmospheric CO_2_through geological time, variation in the background signal caused by the water relationships of C_3_ plants and the taxonomic identity of the C_4_ plants (Tipple & Pagani 2007).

Partial resolution of these issues may come from a new analytical technique for measuring the carbon isotope composition of tiny samples, which currently allows C_3_ or C_4_ pathways to be identified from individual pollen grains with > 85% reliability (Nelson *et al*. 2007). This emerging method should enable researchers to establish directly the photosynthetic pathway of parent plants using grass pollen recovered from sediments dating to the Early Miocene and Oligocene. Unlike analyses of palaeosol or tooth carbon, this technique does not require abundant C_4_ plant biomass to resolve a C_4_ signal from the C_3_ background and offers the potential for identifying rare C_4_ plants in a predominantly C_3_ community, in addition to allowing identification of taxa to at least the family level. Furthermore, because it contrasts ‘pure C_3_’ with ‘pure C_4_’ samples, the technique is not compromised by variations in the isotopic signature of atmospheric CO_2_. Pollen-based evidence may therefore bridge the gap between postulated C_4_ origins and our oldest current evidence for C_4_ plants. However, questions will still remain over the precise taxonomic identity of these plants, and pinning down the earliest C_4_ grasses is only part of the challenge confronting geologists in this field. Plummeting CO_2_ concentrations were correlated with a whole suite of climatic changes during the Oligocene, including falling temperature and increasing aridity (Fig. 3; Zachos *et al*. 2001; Dupont-Nivet *et al*. 2007), and may not have been the only selection pressure for C_4_ photosynthesis.

## Hypothesized drivers of C_4_ grassland expansion

Geological evidence therefore raises two linked questions. If CO_2_ was not the driving force, what caused the Miocene–Pliocene expansion of C_4_ grasslands? And, if C_4_ grasses were present from the Early Miocene onwards, why did they not dominate ecosystems earlier in their evolutionary history? The key to answering both of these questions may lie in fossil evidence that documents an abundance of woody C_3_ plants throughout the Miocene, forming forests, woodlands or savannas in regions that subsequently became C_4_ grasslands (reviewed by Osborne & Beerling 2006). Woody plant cover exerts a major limitation on C_4_ grass abundance, because trees and shrubs rapidly overtop herbaceous plants in the absence of disturbance, and most C_4_ species are intolerant of shading (Sage *et al*. 1999; Sage & Pearcy 2000). Explanations of C_4_ grassland expansion in the geological record therefore evoke a combination of climatic and disturbance factors that reduce tree cover, focusing primarily on changes in rainfall patterns and fire regime.

A number of alternative palaeoclimate hypotheses have been developed. The first is supported by the oxygen isotope signature of palaeosols in South Asia, and proposes that the Miocene replacement of C_3_ woody vegetation by C_4_ grasslands was driven by a strong increase in rainfall seasonality, caused by abrupt intensification of monsoon systems (Table 1; reviewed by Osborne & Beerling 2006). A second hypothesis based on oxygen isotope data from freshwater bivalve shells in Nepal suggests a decrease in the total amount of rainfall with no significant change in seasonality (Dettman *et al*. 2001), and a similar mechanism is evoked for East Africa, rooted in model simulations for the region (Table 1; Sepulchre *et al*. 2006). Both of these climatic drying hypotheses propose that an increased frequency and intensity of drought events killed trees and allowed the incursion of C_4_ grasses, to produce more open savanna or grassland vegetation (Table 1). Both are linked ultimately to tectonic events, including episodes of mountain building such as the uplift of the Tibetan Plateau and East African Rift system, or changes in ocean circulation triggered, for example, by closure of the seaway between the Americas (reviewed by Osborne & Beerling 2006).

**Table 1:** Comparison of the key hypotheses evoked to explain the Miocene–Pliocene expansion of C_4_ grasslands. Alternative hypotheses are not mutually exclusive, and overlap to a significant extent. For example, a change in the fire climate could also be the ultimate reason for an ecotone shift (Keeley & Rundel 2003, 2005).

Hypothesis	Climatic seasonality	Climatic drying	Ecotone shift	Fire climate change
Ultimate reason for Miocene–Pliocene C_4_ grassland expansion	Development of seasonal climates with hot wet season and a dry season	Decrease in total rainfall without significant shifts in seasonality	C_4_ grasses evolved characteristics that allow displacement of woody plants from mesic habitats	Development of seasonal climates promoted fires
Mechanism of C_4_ grassland expansion	Woody plant mortality during annual dry season	Intensified drought events increased woody plant mortality	Shift in the C_4_ grass– woody plant ecotone to wetter (mesic) habitats	Increased frequency of fire prevented woody plant establishment
Why did C_4_ grasses not dominate ecosystems earlier in the Miocene?	Aseasonal climates favoured woody species	Wet climates favoured woody species	C_4_ grasses had not yet evolved characters allowing domination of mesic habitats	Infrequent fires allowed establishment of woody plant communities
Region	South Asia	South Asia, Africa	South Asia, Africa, North America	South Asia, China, West Africa
Key evidence	Palaeosol ^18^O ratios	Freshwater bivalve ^18^O ratios (rainfall seasonality)	Palaeosol structure	Charcoal records
	Monsoon indicators	Model simulations		Analogy with modern ecosystems
References	Quade *et al*. (1989, 1995)	Dettman *et al*. (2001), Sepulchre *et al*. (2006)	Retallack (2001)	Keeley & Rundel (2003, 2005)

**Fig. 4:**
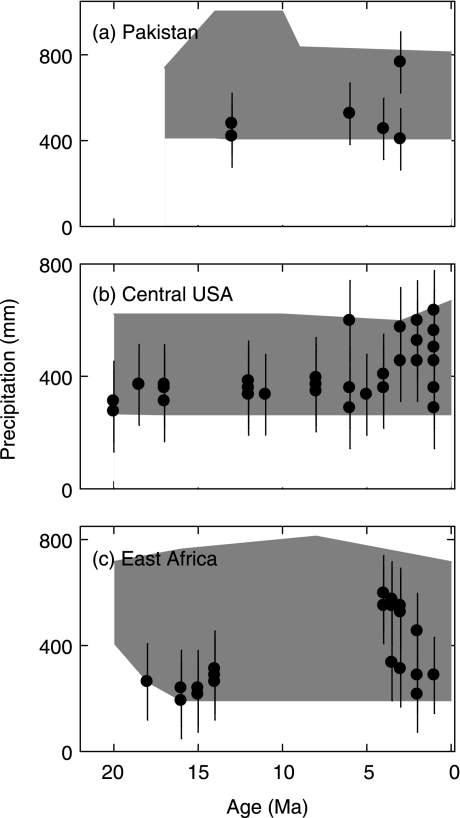
Precipitation estimates based on depth to the calcic horizon in palaeosols from (a) Pakistan, (b) the central USA and (c) East Africa (Retallack 2001). The grey-shaded area denotes the total range of values obtained for all palaeosol types (originating in deserts, grasslands, woodlands and forests), and the symbols show values obtained for mollic palaeosols, which develop only beneath sod-forming grasses (Retallack 2001). These data suggest that grasslands invaded wetter areas from 8 Myr ago (Ma).

An alternative viewpoint has emerged from analyses of palaeosol structure, which use the soil horizon structure to infer a vegetation type of desert, grassland or woodland, and the depth of the calcic horizon to estimate rainfall amounts (Retallack 2001). Application of this approach to geological sediments suggests that spatial gradients in rainfall generated parallel regional variation in vegetation (Fig. 4). Ecosystems varied from desert grassland to dry woodland throughout the Early and Middle Miocene in South Asia, the central and western United States and East Africa (Fig. 4; reviewed by Retallack 2001). Palaeobotanical evidence from East Africa also indicates savanna and woodland vegetation during the Early and Middle Miocene (Jacobs *et al*. 1999). However, the inference of dry climates is not supported by plant macrofossils, phytoliths and pollen from the Great Plains, which indicate productive savanna or woodland vegetation with a significant C_3_ grass component (Jacobs *et al*. 1999; Strömberg 2004, 2005). The presence of palms, gingers and bamboos (Strömberg 2004), woody dicots confined to moister climates today (Axelrod 1985), and giant tortoises and alligators (Hutchinson 1982) suggests a relatively humid, rather than arid, climate. Palaeosol data suggest a major change in the ecology of these ecosystems during the Late Miocene, with C_4_ grasses displacing woodland communities in mesic regions and shifting the grassland–woodland ecotone to higher rainfall areas in North America, Africa and Asia (Table 1, Fig. 4; Retallack 2001). The causes of this ecotone shift are unknown, but have been attributed to the coevolution of grasses and grazers (Retallack 2001), and significant changes in fire regime resulting from shifts in the seasonal distribution, but not total amount, of rainfall (Keeley & Rundel 2005).

Evidence supporting a linkage between fire and the expansion of C_4_ grasslands comes from black carbon (BC) abundance in marine sediments, a geological proxy for fire occurrence. BC increases by 100- to 1000-fold in Pacific Ocean localities downwind of the Indian subcontinent (Herring 1985; Keeley & Rundel 2003), and 5-fold in the South China Sea (Jia *et al*. 2003), during the Miocene–Pliocene interval of major C_4_ grassland expansion in these regions. Sediments off the Atlantic coast of West Africa also show significant increases in charred grass cuticle and pollen abundance during the same period (Morley & Richards 1993). In the last 5 years, a new and compelling hypothesis has therefore added ecosystem-scale feedbacks between fire and vegetation to previous ideas about palaeoclimate (Table 1; Keeley & Rundel 2003, 2005).

Fire sustains C_4_ grasslands by killing woody plants and entrains a positive feedback because the dead foliage of grasses provides abundant fuel for fires, thereby increasing tree mortality and promoting the further spread of grasses (Keeley & Rundel 2003, 2005). The fire hypothesis therefore proposes that increasing climatic seasonality during the Late Miocene raised fire frequency by supporting rapid biomass production and the development of a high fuel load during wet summer conditions. Intensifying winter drought promoted drying, and increased the likelihood that this material would ignite, with ignition itself provided by lightening strikes at the end of the dry season. Further feedbacks on the fire regime have been proposed via the hydrological cycle (Beerling & Osborne 2006) and wind strength (Tipple & Pagani 2007), but these remain speculative at present.

Ecosystem fire regimes show an important interaction with climate. Fuel remains too moist to support frequent fires in wet, aseasonal environments, whilst the low productivity of dry climate regions produces too little fuel to carry significant fires (Keeley & Rundel 2005). However, given sufficient periods of dry weather, fire has the potential to displace mesic forests in favour of grasslands. This is demonstrated in modern ecosystems by complementary data from: model simulations showing that fire reduces forest cover by ∼50% at the global scale (Bond *et al*. 2005); analysis of woody plant cover across Africa indicating that significant areas of savanna are maintained by disturbance in regions with sufficient rainfall to support forest (Sankaran *et al*. 2005); fire exclusion experiments which allow the establishment of fire-sensitive trees in mesic savanna ecosystems (Bond *et al*. 2003, 2005); and the fire-mediated replacement of native forests on Pacific Islands by invasive grasses (D’Antonio & Vitousek 1992). These observations each provide indirect support for the hypothesized mechanism of vegetation change at the Miocene–Pliocene boundary.

An emerging consensus therefore evokes interactions between palaeoclimate change, vegetation–climate relationships and fire frequency to explain the Miocene expansion of C_4_ grasslands (Table 1; Keeley & Rundel 2005). This ‘consensus scenario’ suggests that Early and Middle Miocene landscapes were dominated by C_3_ forests or woodlands, with C_4_ grasses occupying open ground between the patches of woody plants. Increasing seasonality during the Late Miocene concentrated rainfall into a hot growing season, creating a fire regime that removed woody vegetation and shifted the woodland–grassland ecotone to wetter areas (Table 1). Formulated in this way, the hypothesis asserts that climates capable of supporting frequently burning mesic C_4_ grasslands at the expense of woodland and forests were absent during the Early and Middle Miocene.

However, application of the hypothesis to North and South America is problematic in two important respects. First, patterns of climatic change in these regions at the Miocene–Pliocene boundary are less clear than those in Asia, and an increase in rainfall seasonality has yet to be demonstrated (Passey *et al*. 2002; Fox & Koch 2004; Osborne & Beerling 2006). Secondly, pollen records, phytolith assemblages and mammalian tooth morphology suggest that a transition from closed forest to open woody vegetation with a significant C_3_ grass component may have occurred as early as 25 Ma (Late Oligocene to Early Miocene) in the Great Plains (Strömberg 2004, 2005) and 32 Ma (Early Oligocene) in South America (Jacobs *et al*. 1999). The adaptive radiation of specialist grazers (Strömberg 2006) and an extremely high browser diversity in these Great Plains ecosystems resulted in species-rich mammalian herbivore communities by 15 Ma (Janis *et al*. 2000). Carbon isotope data demonstrate that some grazers showed a dietary shift in the Late Miocene, subsisting almost exclusively on C_4_ plants for the 2 Myr before C_4_ grassland expansion, with presumably significant, but currently unknown, effects on grass–tree dynamics (Fox & Koch 2004; Beerling & Osborne 2006).

I argue that our overall understanding and ability to test climatic and ecological hypotheses regarding Miocene ecosystems would be improved by an explicit consideration of C_4_ grass phylogeny. To make this case, I first present biogeographical data indicating significant contrasts in the abundance of different C_4_ grass clades along rainfall gradients, and show how this information might be used in hypothesis testing. Secondly, I re-analyse a published data set in the same context, suggesting that changes in the species richness of independent C_4_ grass lineages track the length of the rainy season. Finally, I present intriguing preliminary evidence of fire controls on the abundance of particular C_4_ clades.

## Contrasting climate relationships of C_4_ grass clades

**Fig. 5:**
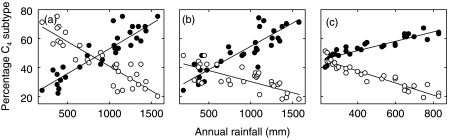
(a) Percentage of C_4_ grasses in US floras belonging to the Panicoideae (•) and the Chloridoideae ○) subfamilies (Taub 2000). (b) Percentage of C_4_ grasses in floras with the NADP-ME (•) and NAD-ME (○) subtypes in the USA (Taub 2000) and (c) Australia (Hattersley 1992). Because each flora covers a differing geographical area, values are expressed as a percentage to normalize for species–area biases. (a) and (b) express the same data set on a different basis, excluding (a) members of the Aristidoideae, and (b) species with the PCK subtype of C_4_ photosynthesis.

The subfamilies Panicoideae and Chloridoideae together account for the majority of modern C_4_ grass species (Fig. 1; Linder & Rudall 2005), but analyses of regional biodiversity patterns suggest a crucial difference in the climate relationships of these major clades. For the United States and Argentina, the percentage of C_4_ species belonging to the Panicoideae shows a positive correlation with annual rainfall, whereas the same relationship for the Chloridoideae is negative (Fig. 5; Taub 2000; Cabido *et al*. 2007).

The mechanisms underpinning these relationships are unclear at present, but may be linked to the close association between biochemical subtypes of C_4_ photosynthesis and specific phylogenetic groups; the majority of Panicoideae species utilize the NADP-malic enzyme (NADP-ME) pathway whereas most members of the Chloridoideae use the NAD-malic enzyme (NAD-ME) pathway (Taub 2000). As a consequence, contrasting correlations with rainfall are also recognized for different C_4_ subtypes (Fig. 5b,c; e.g. Hattersley 1992; Taub 2000; Cabido *et al*. 2007). Experimental investigations suggest that these alternative forms of the pathway may be differentially adaptive in relation to water availability (Ghannoum *et al*. 2002), or a correlated environmental variable such as soil nutrient status (Ghannoum *et al*. 2005). However, none of these experiments have explicitly controlled for phylogeny, and the issue remains unresolved. Further questions remain about how these species richness patterns translate into plant abundance along rainfall gradients.

Despite these uncertainties, the geographical distributions of modern C_4_ grasses indicate that changing rainfall patterns will drive significant shifts in the phylogenetic composition of C_4_ grass communities. This observation has important implications for palaeoclimate hypotheses about the Miocene–Pliocene expansion of C_4_ grasslands, because it allows predictions about the clades of C_4_ grasses that are involved. First, the range of Middle Miocene ecosystems along inferred rainfall gradients (Retallack 2001) should contain varying proportions of C_4_ grass subfamilies, following the qualitative patterns in Fig. 5(a). Secondly, a shift in the woodland–grassland ecotone to higher rainfall areas should increase the proportion of C_4_ species belonging to the Panicoideae relative to the Chloridoideae. Finally, a decrease in the total amount of rainfall should drive the reverse pattern, favouring species of the Chloridoideae relative to the Panicoideae.

These opposing predictions are especially critical for regions such as East Africa and South Asia, where alternative hypotheses are postulated. Crucially, they can be tested by using emerging techniques for quantifying the relative abundance of different grass subfamilies within fossil phytolith assemblages (e.g. Prasad *et al*. 2005), providing a new means of evaluating alternative hypotheses using geological evidence. Conversely, the use of these techniques to reconstruct the phylogenetic make-up of Miocene grassland communities in the Great Plains could offer vital clues about the environmental drivers of C_4_ grassland expansion in this region. Finally, quantifying shifts in the abundance of different grass subfamilies could aid in the interpretation of carbon isotope signals. For example, a decrease in the proportion of Chloridoideae (NAD-ME) relative to Panicoideae (NADP-ME) species would be expected to ‘amplify’ the C_4_ signal, because discrimination against ^13^C is stronger in NAD-ME than NADP-ME grasses (Hattersley 1982), i.e. the NAD-ME type is slightly more ‘C_3_-like’. The power of these approaches would be increased greatly by a better understanding of how C_4_ grass distributions vary in relation to rainfall seasonality and fire frequency.

## Re-evaluating the role of seasonal rainfall

Previous studies suggest that species richness of the tribes Andropogoneae and Paniceae (Fig. 1; Panicoideae) may change significantly in response to the intensity and seasonality of rainfall. For southern Africa, Gibbs-Russell (1988) used a qualitative comparison of floristic and climatic data to show that Andropogoneae species account for the largest fraction of the Panicoideae in monsoonal summer rainfall areas where mean annual precipitation (MAP) > 500 mm. The opposite pattern occurs in the Paniceae, which account for the largest proportion of Panicoideae species in more arid summer-rainfall regions (MAP < 500 mm).

Hartley (1950, 1958a, b) carried out a similar analysis at the global scale, but expressed species numbers in each tribe as a fraction of the total grass flora, rather than restricting the analysis to the subfamily Panicoideae. Based on qualitative descriptions of maps, Hartley hypothesized that centres of the highest species diversity in the Andropogoneae are typically located in subtropical monsoon climates with a short rainy season (Hartley 1958a), whereas the highest diversity in the Paniceae occurs in less seasonal wet equatorial climates (Hartley 1958b). To evaluate this hypothesis further, I have carried out a quantitative analysis of the same species richness data sets (Hartley 1950, 1958a) by using climate data recorded at nearby meteorological stations (Müller 1982). A series of generalized linear models (GLIMs) were fitted to the data, using a quasibinomial distribution in place of the binomial distribution when data were underdispersed [glm(), R version 2.4.1, The R Foundation for Statistical Computing]. I used a forward stepwise approach, testing first the simple additive hypotheses posed in the literature, and then adding more variables and interactions.

**Fig. 6:**
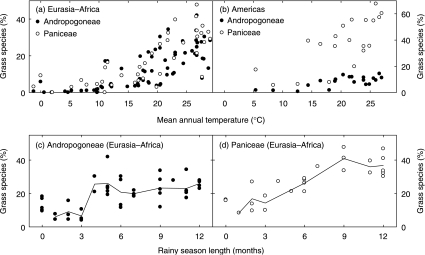
Global variation in relative species richness within the grass subfamily Panicoideae, showing species abundance within the tribes Andropogoneae and Paniceae as a proportion of all grass species in a flora (%), relative to climatic variables. The upper panels show variation due to mean annual temperature for (a) Eurasia and Africa and (b) North and South America, and the lower panels show variation due to rainy season length (RSL) in Eurasia and Africa for (c) the Andropogoneae and (d) the Paniceae, with lines indicating mean monthly values.

Numerous previous studies have demonstrated that temperature is the primary global control on species richness for C_4_ grasses (reviewed by Sage *et al*. 1999), and the Andropogoneae and Paniceae tribes followed this general pattern (Table 2a,b). GLIMs for Eurasia–Africa and the Americas showed highly significant additive effects of mean annual temperature (MAT) and tribe on relative species richness (Fig. 6a,b, Table 2a,b). Relative diversity was lower in the Andropogoneae than the Paniceae (Table 2a,b), and this difference was more pronounced in the Americas than Eurasia and Africa (Fig. 6a,b).

**Table 2:** Generalized linear models (GLIMs) explaining the global variation in relative species richness within the grass subfamily Panicoideae. The models consider the interacting effects of mean annual temperature (MAT), rainy season length (RSL) and tribe (Andropogoneae or Paniceae).

Factor	d.f.	F	P
(a) Americas			
MAT	1,38	35.9	< 0.0001
Tribe	1,37	120.6	< 0.0001
MAT × Tribe	–	–	NS
(b) Eurasia–Africa			
MAT	1,112	170.2	< 0.0001
Tribe	1,111	8.1	0.0053
MAT × Tribe	–	–	NS
(c) Eurasia-Africa			
MAT	1,59	40.1	< 0.0001
RSL	1,58	25.4	< 0.0001
Tribe	1,57	6.1	0.0162
MAT × Tribe	–	–	NS
RSL × Tribe	–	–	NS
(d) Eurasia–Africa			
MAT	1,59	61.4	< 0.0001
RSL (binned)	1,57	31.8	< 0.0001
Tribe	1,56	12.7	0.0008
RSL × Tribe	2,54	3.1	0.0549
MAT × Tribe	–	–	NS

I then added rainy season length (RSL) to the model, restricting the analysis to tropical and subtropical localities (latitude < 35°N or S). Because the latitude criterion excluded most of the data from the Americas, this analysis focused on Eurasia and Africa. RSL was estimated for each locality using a biologically based definition of the growing season for C_4_ grasses; as the number of consecutive months in which the mean minimum temperature > 10 °C and total precipitation > 30 mm (Collatz *et al*. 1998). The temperature threshold for growth was based upon two lines of complementary evidence: (i) experimental observations of chilling-mediated photoinhibition and impairment of leaf extension in C_4_ grasses (Long 1983); and (ii) the minimum summer temperature required for C_4_ species to persist in a grass flora (Sage *et al*. 1999), a threshold that was observed in this data set. These criteria defined a reasonable (non-zero) rainy season for all localities except those in desert regions (e.g. Syria and Yemen), where plants are probably associated with bodies of water or infrequent rains, or at high elevation (e.g. Lesotho), where C_4_ species may show an unusual resistance to low temperature extremes (Márquez *et al*. 2007). These localities with a zero RSL were excluded from further analysis.

**Fig. 7:**
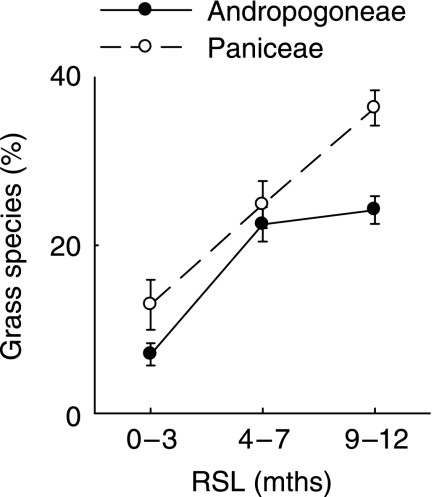
Interaction between species richness in the Andropogoneae and Paniceae tribes and rainy season length (RSL) in Eurasia and Africa. Species abundance within each tribe is expressed as a proportion of all grass species in a local flora (%), and shown as the mean ± SE for each RSL category.

The minimum adequate GLIM showed highly significant additive effects of MAT, RSL and tribe, but not the hypothesized interaction between MAT and RSL (Table 2c). Species richness increased with RSL in both tribes, but showed a differing nonlinear response: values for the Andropogoneae were low when 0 < RSL ≤ 3 months, and high when 4 ≤ RSL ≤ 12 months (Fig. 6c); in contrast, values for the Paniceae increased in an approximately linear response to reach a maximum at RSL ≥ 9 months (Fig. 6d). I therefore constructed a new GLIM attempting to account for these apparent nonlinearities by grouping RSL data into three temporal categories: 1–3, 4–7 and 9–12 months (there were no localities with RSL = 8 months). This model showed an additive effect of MAT, and an interaction between RSL and tribe (Table 2d). The interaction was caused by equal species richness in climates with a rainy season of 7 months or less, and greater species richness in the Panicoideae than Andropogoneae in aseasonal climates with rainy seasons of 9 months or more (Fig. 7).

This quantitative analysis supports the hypothesis that species richness in the Paniceae is highest in moist, aseasonal climates of the subtropics and tropics, but fails to show a similar association between maximal Andropogoneae diversity and a short rainy season. Instead, species richness in the Andropogoneae remains constant across localities with rainy seasons of 4–12 months (Figs 6c and 7). The analysis therefore highlights a phylogenetic contrast between the tribes that could prove useful for hypothesis testing in the geological record: a climatic change from moist, aseasonal conditions to a strongly seasonal climate is expected to drive a shift in the grass flora from a predominance of Paniceae species towards a more equal representation of the Paniceae and Andropogoneae. Further work is required before this expectation can be tested in the fossil record, because techniques for analysing phytolith assemblages cannot yet distinguish tribes of the Panicoideae. However, archaeologists already use phytolith-based diagnostics for identifying individual crop species of the Andropogoneae (Piperno 2006), indicating good promise for increasing the taxonomic resolution of this technique.

The value of these observed patterns of species richness would be increased greatly by an understanding of the underlying mechanisms. One possibility is suggested by Bond *et al*. (2003), who hypothesize that the Andropogoneae are key to the fire-mediated displacement of forests by grasslands in mesic climates of southern Africa. These authors note the dominance of this group in fire-maintained and nutrient-poor mesic savannas, the obligate dependence of Andropogoneae species such as *Themeda triandra* on frequent defoliation, and their decline in the absence of fires. Frequent fires are promoted by the rapid growth of Andropogoneae species during summer, the accumulation of tannin-like compounds in their foliage, and the low nutritional quality of leaves during winter (reviewed by Bond *et al*. 2003). These latter traits retard decomposition and result in a low palatability to grazers during the dry season, leading to the build up of a high fuel load for fires.

The evolution of such fire-promoting traits in one or more clades of C_4_ grasses during the Miocene therefore provides a possible explanation for how the grassland–woodland ecotone shifted to more mesic areas. But what might have selected for these characters? One idea is suggested by the Miocene radiation of grazers, and the specialization of some animals on a C_4_ diet prior to grassland expansion. Recent studies indicate that modern grasses have evolved a suite of characteristics to resist grazing, including the accumulation of phenolic compounds (e.g. tannins), a high silica content, and tough fibrous leaves with a low palatability and nutritional quality (Burt-Smith *et al*. 2003; Massey *et al*. 2007). Ecological theory proposes that selection for these traits is strongest in nutrient-poor habitats (Grime 2001), and empirical evidence shows that grazer-driven selection for unpalatable species occurs in dry ecosystems with a long history of herbivory (Díaz *et al*. 2007). Speculative links may therefore be drawn between high grazing pressure in nutrient-poor savannas during the Miocene, and selection for grazing-resistant (but fire-promoting) traits in C_4_ grasses. As these traits may also occur in C_3_ grasses (Grigulis *et al*. 2005), this hypothesis provides an ecological explanation for Miocene C_4_ grassland expansion that is not directly linked to photosynthetic pathway.

## Conclusions

The origin of C_4_ photosynthesis and expansion of C_4_ grasslands were major events in Earth history with significant consequences for tropical and subtropical ecology. Newly uncovered geological evidence has bolstered support for decreasing CO_2_ as a selection agent for the pathway, leading to the expectation that C_4_ grasses first evolved during the Oligocene. However, these same data cast serious doubt on the role of CO_2_ in C_4_ grassland expansion. Instead, current ecological understanding of vegetation–fire dynamics in savannas has generated hypotheses integrating the roles of climate change and fire. I propose that the contrasting modern diversity patterns shown by independent C_4_ grass clades along climatic gradients generate a further expectation: that palaeoclimate change forced significant shifts in the phylogenetic composition of Miocene grass communities. New techniques for the geochemical analysis of pollen and the extraction of phylogenetic information from phytolith assemblages provide the necessary tools for testing these ideas, and will help to bring the evolutionary history of C_4_ grasses into sharper focus.

## References

[b1] Aliscioni SS, Giussani LM, Zuloaga FO, Kellogg EA (2003). A molecular phylogeny of *Panicum* (Poaceae: Paniceae): tests of monophyly and phylogenetic placement within the Panicoideae. American Journal of Botany.

[b2] Axelrod DI (1985). Rise of the grassland biome, central North America. Botanical Review.

[b3] Beerling DJ, Osborne CP (2006). The origin of the savanna biome. Global Change Biology.

[b4] Berner RA (1998). The carbon cycle and CO_2_ over Phanerozoic time: the role of land plants. Philosophical Transactions of the Royal Society of London B.

[b5] Bond WJ, Midgley GF, Woodward FI (2003). What controls South African vegetation – climate or fire?. South African Journal of Botany.

[b6] Bond WJ, Woodward FI, Midgley GF (2005). The global distribution of ecosystems in a world without fire. New Phytologist.

[b7] Burt-Smith GS, Grime JP, Tilman D (2003). Seedling resistance to herbivory as a predictor of relative abundance in a synthesised prairie ecosystem. Oikos.

[b8] Cabido M, Pons E, Cantero JJ, Lewis JP, Anton A (2007). Photosynthetic pathway variation among C_4_ grasses along a precipitation gradient in Argentina. Journal of Biogeography.

[b9] Cerling TE, Sage RF, Monson RK (1999). Palaeorecords of C_4_ plants and ecosystems. C_4_ Plant Biology.

[b10] Cerling TE, Harris JM, MacFadden BJ, Leakey MG, Quade J, Eisenmann V, Ehleringer JR (1997). Global vegetation change through the Miocene/Pliocene boundary. Nature.

[b11] Cerling TE, Quade J, Wang Y (1994). Expansion and emergence of C4 plants. Nature.

[b12] Collatz GJ, Berry JA, Clark JS (1998). Effects of climate and atmospheric CO_2_ partial pressure on the global distribution of C_4_ grasses: past, present, and future. Oecologia.

[b13] D’Antonio CM, Vitousek PM (1992). Biological invasions by exotic grasses, the grass/fire cycle, and global change. Annual Review of Ecology and Systematics.

[b14] Dettman DL, Kohn MJ, Quade J, Ryerson FJ, Ojha TP, Hamidullah S (2001). Seasonal stable isotope evidence for a strong Asian monsoon throughout the past 10.7 m.y. Geology.

[b15] Díaz S, Lavorel S, McIntyre S, Falczuk V, Casanoves F, Milchunas DG, Skarpe C, Rusch G, Sternberg M, Noy-Meir I, Landsberg J, Zhang W, Clark H, Campbell BD (2007). Plant trait responses to grazing – a global synthesis. Global Change Biology.

[b16] Dupont-Nivet G, Krijgsman W, Langereis CG, Abels HA, Dai S, Fang X (2007). Tibetan plateau aridification linked to global cooling at the Eocene–Oligocene transition. Nature.

[b17] Ehleringer JR, Cerling TE, Helliker BR (1997). C_4_ photosynthesis, atmospheric CO_2_, and climate. Oecologia.

[b18] Ehleringer JR, Sage RF, Flanagan LB, Pearcy RW (1991). Climate change and the evolution of C_4_ photosynthesis. Trends in Ecology and Evolution.

[b19] Fox DL, Koch PL (2003). Tertiary history of C_4_ biomass in the Great Plains, USA. Geology.

[b20] Fox DL, Koch PL (2004). Carbon and oxygen isotope variability in Neogene paleosol carbonates: constraints on the evolution of the C_4_-grasslands of the Great Plains, USA. Palaeogeography, Palaeoclimatology, Palaeoecology.

[b21] Gaut BS, Doebley JF (1997). DNA sequence evidence for the segmental allotetraploid origin of maize. Proceedings of the National Academy of Sciences of the USA.

[b22] Ghannoum O, Evans JR, Chow WS, Andrews TJ, Conroy JP, von Caemmerer S (2005). Faster Rubisco is the key to superior nitrogen-use efficiency in NADP-malic enzyme relative to NAD-malic enyme C_4_ grasses. Plant Physiology.

[b23] Ghannoum O, von Caemmerer S, Conroy JP (2002). The effect of drought on plant water use efficiency of nine NAD-ME and nine NADP-ME Australian C_4_ grasses. Functional Plant Biology.

[b24] Gibbs-Russell GE (1988). Distribution of subfamilies and tribes of Poaceae in southern Africa. Monographs in Systematic Botany from the Missouri Botanical Garden.

[b25] Giussani LM, Cota-Sanchez JH, Zuloaga FO, Kellogg EA (2001). A molecular phylogeny of the grass subfamily Panicoideae (Poaceae) shows multiple origins of C_4_ photosynthesis. American Journal of Botany.

[b26] Grass Phylogeny Working Group (2001). Phylogeny and sub-familial classification of the grasses (Poaceae). Annals of the Missouri Botanical Garden.

[b27] Grigulis K, Lavorel S, Davies ID, Dossantos A, Llorets F, Villa M (2005). Landscape-scale positive feedbacks between fire and expansion of the large tussock grass, *Ampelodesmos mauritanica*. Catalan shrublands. Global Change Biology.

[b28] Grime JP (2001). Plant Strategies, Vegetation Processes, and Ecosystem Properties.

[b29] Hartley W (1950). The global distribution of tribes of the Gramineae in relation to historical and environmental factors. Australian Journal of Agricultural Research.

[b30] Hartley W (1958a). Studies on the origin, evolution, and distribution of the Gramineae. I. The tribe Andropogoneae. Australian Journal of Botany.

[b31] Hartley W (1958b). Studies on the origin, evolution, and distribution of the Gramineae. II. The tribe Paniceae. Australian Journal of Botany.

[b32] Hartley W (1961). Studies on the origin, evolution, and distribution of the Gramineae. IV. The genus *Poa* L. Australian Journal of Botany.

[b33] Hartley W (1973). Studies on the origin, evolution, and distribution of the Gramineae. V. The subfamily Festucoideae. Australian Journal of Botany.

[b34] Hartley W, Slater C (1960). Studies on the origin, evolution, and distribution of the Gramineae. III. The tribes of the subfamily Eragrostoideae. Australian Journal of Botany.

[b35] Hattersley PW (1982). δ^13^C values of C_4_ types in grasses. Australian Journal of Plant Physiology.

[b36] Hattersley PW, Chapman GP (1992). C_4_ photosynthetic pathway variation in grasses (Poaceae): its significance for arid and semi-arid lands. Desertified Grasslands: Their Biology and Management.

[b37] Herring JR, Sundquist ET, Broecker WS (1985). Charcoal fluxes into sediments of the North Pacific Ocean: the Cenozoic record of burning. The Carbon Cycle and Atmospheric CO_2_: Natural Variations Archean to Present.

[b38] Hodson MJ, White PJ, Mead A, Broadley MR (2005). Phylogenetic variation in the silicon composition of plants. Annals of Botany.

[b39] Hutchinson JH (1982). Turtle, crocodilian, and champosaur diversity changes in the Cenozoic of the north-central region of western United States. Palaeogeography, Palaeoclimatology, Palaeoecology.

[b40] Jacobs BF, Kingston JD, Jacobs LL (1999). The origin of grass-dominated ecosystems. Annals of the Missouri Botanical Garden.

[b41] Janis CM, Damouth J, Theodor JM (2000). Miocene ungulates and terrestrial primary productivity: where have all the browsers gone?. Proceedings of the National Academy of Sciences of the USA.

[b42] Jia G, Peng P, Zhao Q, Jian Z (2003). Changes in terrestrial ecosystem since 30 Ma in East Asia: stable isotope from black carbon in the South China Sea. Geology.

[b43] Keeley JE, Rundel PW (2003). Evolution of CAM and C_4_ carbon-concentrating mechanisms. International Journal of Plant Science.

[b44] Keeley JE, Rundel PW (2005). Fire and the Miocene expansion of C_4_ grasslands. Ecological Letters.

[b45] Kellogg EA, Sage RF, Monson RK (1999). Phylogenetic aspects of the evolution of C_4_ photosynthesis. C_4_ Plant Biology.

[b46] Linder HP, Rudall PJ (2005). Evolutionary history of Poales. Annual Review of Ecology and Systematics.

[b47] Lloyd J, Farquhar GD (1994). ^13^C discrimination during CO_2_ assimilation by the terrestrial biosphere. Oecologia.

[b48] Long SP (1983). C_4_ photosynthesis at low temperatures. Plant Cell Environment.

[b49] Márquez EJ, Rada F, Fariñas MR (2007). Freezing tolerance in grasses along an altitudinal gradient in the Venezuelan Andes. Oecologia.

[b50] Massey FP, Ennos AR, Hartley SE (2007). Grasses and the resource-availability hypothesis: the importance of silica-based defences. Journal of Ecology.

[b51] Morgan ME, Kingston JD, Marino BD (1994a). Carbon isotopic evidence for the emergence of C4 plants in the Neogene from Pakistan and Kenya. Nature.

[b52] Morgan ME, Kingston JD, Marino BD (1994b). Reply. Expansion and emergence of C4 plants. Nature.

[b53] Morley RJ, Richards K (1993). Gramineae cuticle: a key indicator of Late Cenozoic climatic change in the Niger Delta. Review of Palaeobotany and Palynology.

[b54] Müller MJ (1982). Selected Climatic Data for a Global Set of Standard Stations for Vegetation Science.

[b55] Nelson DM, Hu FS, Mikucki JA, Tian J, Pearson A (2007). Carbon-isotopic analysis of individual pollen grains from C_3_ and C_4_ grasses using a spooling wire microcombustion interface. Geochemica et Cosmochemica Acta.

[b56] Osborne CP, Beerling DJ (2006). Nature's green revolution: the remarkable evolutionary rise of C_4_ plants. Philosophical Transactions of the Royal Society of London B.

[b57] Pagani M, Freeman KH, Arthur MA (1999). Late Miocene atmospheric CO_2_ concentrations and the expansion of C_4_ grasses. Science.

[b58] Pagani M, Zachos J, Freeman KH, Tipple B, Boharty S (2005). Marked decline in atmospheric carbon dioxide concentrations during the Paleogene. Science.

[b59] Passey BH, Cerling TE, Perkins ME, Voorhies MR, Harris JM, Tucker ST (2002). Environmental change in the Great Plains: an isotopic record from fossil horses. Journal of Geology.

[b60] Pearson PN, Palmer MR (2000). Atmospheric carbon dioxide concentrations over the past 60 million years. Nature.

[b61] Piperno DR (1988). Phytolith Analysis, an Archaeological and Geological Perspective.

[b62] Piperno DR (2006). Phytoliths: a Comprehensive Guide for Archaeologists and Paleoecologists.

[b63] Piperno DR, Sues H-D (2005). Dinosaurs dined on grass. Science.

[b64] Prasad V, Strömberg CAE, Alimohammadian H, Sahni A (2005). Dinosaur coprolites and the early evolution of grasses and grazers. Science.

[b65] Pulquiéro MJF, Nichols RA (2007). Dates from the molecular clock: how wrong can we be?. Trends in Ecology and Evolution.

[b66] Quade J, Cater JML, Ojha TP, Adam J, Harrison TM (1995). Late Miocene environmental change in Nepal and the northern Indian subcontinent: stable isotope evidence from paleosols. GSA Bulletin.

[b67] Quade J, Cerling TE (1995). Stable isotopes in paleosols and the expansion of C_4_ grasses in the late Miocene of Northern Pakistan. Palaeogeography, Palaeoclimatology, Palaeoecology.

[b68] Quade J, Cerling TE, Bowman JR (1989). Development of the Asian monsoon revealed by marked ecological shift during the latest Miocene in northern Pakistan. Nature.

[b69] Retallack GJ (2001). Cenozoic expansion of grasslands and climatic cooling. Journal of Geology.

[b70] Royer DL (2006). CO_2_-forced climate thresholds during the Phanerozoic. Geochemica et Cosmochemica Acta.

[b71] Royer DL, Wing SL, Beerling DJ, Jolley DW, Koch PL, Hickey LJ, Berner RA (2001). Paleobotanical evidence for near present-day levels of atmospheric CO_2_ during part of the Tertiary. Science.

[b72] Sage RF (2004). The evolution of C_4_ photosynthesis. New Phytologist.

[b73] Sage RF, Pearcy RW, Leegood RC, Sharkey TD, von Caemmerer S (2000). The physiological ecology of C_4_ photosynthesis. Photosynthesis: Physiology and Metabolism.

[b74] Sage RF, Wedin DA, Li M, Sage RF, Monson RK (1999). The biogeography of C_4_ photosynthesis: patterns and controlling factors. C_4_ Plant Biology.

[b75] Sánchez-Ken JG, Clark LG, Kellogg EA, Kay EE (2007). Reinstatement and emendation of Subfamily Micrairoideae (Poaceae). Systematic Botany.

[b76] Sankaran M, Hanan NP, Scholes RJ (2005). Determinants of woody cover in African savannas. Nature.

[b77] Sepulchre P, Ramstein G, Fluteau F, Schuster M, Tier JJ, Brunet M (2006). Tectonic uplift and eastern African aridification. Science.

[b78] Smith FA (2001). Phytolith carbon isotope records of Neogene grasses. GSA Abstracts with Programs.

[b79] Smith FA, White JWC (2004). Modern calibration of phytolith carbon isotope signature for C_3_/C_4_ paleograssland reconstruction. Palaeogeography, Palaeoclimatology, Palaeoecology.

[b80] Strömberg CAE (2004). Using phytolith assemblages to reconstruct the origin and spread of grass-dominated habitats in the great plains of North America during the late Eocene to early Miocene. Palaeogeography, Palaeoclimatology, Palaeoecology.

[b81] Strömberg CAE (2005). Decoupled taxonomic radiation and ecological expansion of open-habitat grasses in the Cenozoic of North America. Proceedings of the National Academy of Sciences of the USA.

[b82] Strömberg CAE (2006). Evolution of hypsodonty in equids: testing a hypothesis of adaptation. Paleobiology.

[b83] Taub DR (2000). Climate and the U.S. distribution of C_4_ grass subfamilies and decarboxylation variants of C_4_ photosynthesis. American Journal of Botany.

[b84] Tipple BJ, Pagani M (2007). The early origins of terrestrial C_4_ photosynthesis. Annual Review of Earth and Planetary Science.

[b85] Watson L, Dallwitz MJ, The Grass Genera of the World: Descriptions, Illustrations, Identification, and Information Retrieval; Including Synonyms, Morphology, Anatomy, Physiology, Phytochemistry, Cytology, Classification, Pathogens, World and Local Distribution, and References (1992). http://delta-intkey.com.

[b86] Zachos J, Pagani M, Sloan L, Thomas E, Billups K (2001). Trends, rhythms, and aberrations in global climate 65 Ma to present. Science.

